# Diagnosis of Phantom Abdominal Tumors

**Published:** 1871-10

**Authors:** 


DIAGNOSIS OF PHANTOM ABDOMIMAL TUMORS.—In an interesting clinical lecture published in the *Medical Times*, Dr. J. M. DeCaster states that the most effcient mode of diagnosticating phantom tumors of the abdomen is by the use of an anæsthetic. When under the influence of ether, a phantom tumor must disappear, and thus remove all doubt as to the nature of the case. He says, " not in a single instance have I found these apparent tumors remain when the patient is under the influence of ether or chloroform, though they reappear, and quickly, when he passes from under its influence." Thus, we can control the diagnosis not only of phantom tumors, but muscular contractions of the abdomen, not unfrequently feigned, and which high authority declares a source of difficulty almost impossible to get over."

